# Supplementary material for: Genome-Wide Analysis of C/S1-bZIP Subfamilies in Populus tomentosa and Unraveling the Role of PtobZIP55/21 in Response to Low Energy
Source: Int J Mol Sci. 2024 May 9;25(10):5163. doi: 10.3390/ijms25105163 (PMC11120861; doi:10.3390/ijms25105163)
Supplement: Supplementary file 1 [file ijms-25-05163-s001.zip › ijms-2971733-supplementary/Supplementary tables.pdf]

Table S1. *C/S1-bZIP* genes identified in *P. tomentosa*.

Gene names	Gene ID	Chr	Number of Amino Acid (aa)	Molecular Weight (kDa)	Theoretical pI	Instability Index
PtobZIP42	*POTOM_026607*	6	301	32.48	5.72	53.91
PtobZIP25	*POTOM_020647*	5	457	49.37	6.36	61.39
PtobZIP32	*POTOM_021892*	5	443	47.74	6.69	54.87
PtobZIP7	*POTOM_009536*	2	497	53.85	5.78	57.58
PtobZIP49	*POTOM_031535*	8	143	16.46	9.36	68.21
PtobZIP63	*POTOM_038208*	10	143	16.51	9.66	56.33
PtobZIP75	*POTOM_048317*	14	139	15.88	7.84	58.38
PtobZIP15	*POTOM_010759*	2	144	16.50	8.88	55.79
PtobZIP44	*POTOM_027938*	7	164	18.71	6.2	62.87
PtobZIP27	*POTOM_021242*	5	162	18.25	6.59	70.33
PtobZIP21	*POTOM_017716*	4	155	17.44	8.1	61.51
PtobZIP55	*POTOM_034782*	9	155	17.30	8.09	47.95
PtobZIP33	*POTOM_022015*	5	158	18.32	6.05	47.9
PtobZIP6	*POTOM_009428*	2	156	17.90	6.18	43.91

Table S2. Accession numbers of *C/S1*-genes presented in Fig. 1.

Gene Name	Locus names or Accession nos. or AGI codes	Species
AtbZIP9	*AT5G24800*	*Arabidopsis thaliana*
AtbZIP10	*AT4G02640*	*A. thaliana*
AtbZIP25	*AT3G54620*	*A. thaliana*
AtbZIP63	*AT5G28770*	*A. thaliana*
AtbZIP1	*AT5G49450*	*A. thaliana*
AtbZIP2	*AT2G18160*	*A. thaliana*
AtbZIP11	*AT4G34590*	*A. thaliana*
AtbZIP44	*AT1G75390*	*A. thaliana*
AtbZIP53	*AT3G62420*	*A. thaliana*
MdbZIP81	*MDP0000431572*	*Malus domestica*
MdbZIP82	*MDP0000441891*	*M. domestica*
MdbZIP92	*MDP0000680042*	*M. domestica*
MdbZIP75	*MDP0000270365*	*M. domestica*
MdbZIP24	*MDP0000275309*	*M. domestica*
MdbZIP56	*MDP0000176747*	*M. domestica*
MdbZIP2	*MDP0000249561*	*M. domestica*
MdbZIP3	*MDP0000265875*	*M. domestica*
MdbZIP41	*MDP0000205823*	*M. domestica*
MdbZIP61	*MDP0000239026*	*M. domestica*
MdbZIP40	*MDP0000521934*	*M. domestica*
MdbZIP73	*MDP0000448715*	*M. domestica*
MdbZIP20	*MDP0000437680*	*M. domestica*
MdbZIP63	*MDP0000234798*	*M. domestica*
MdbZIP64	*MDP0000949327*	*M. domestica*
SlbZIP06	*Solyc01g097330*	*Solanum lycopersicum*
SlbZIP48	*Solyc08g006110*	*S. lycopersicum*
SlbZIP49	*Solyc08g022080*	*S. lycopersicum*
SlbZIP04	*Solyc01g079480*	*S. lycopersicum*
SlbZIP07	*Solyc01g100460*	*S. lycopersicum*
SlbZIP10	*Solyc01g109880*	*S. lycopersicum*
SlbZIP15	*Solyc02g084860*	*S. lycopersicum*
SlbZIP19	*Solyc03g033730*	*S. lycopersicum*
SlbZIP34	*Solyc04g080740*	*S. lycopersicum*
SlbZIP39	*Solyc06g009640*	*S. lycopersicum*
StbZIP38	*PGSC0003DMT400038493*	*Solanum tuberosum*
StbZIP02	*PGSC0003DMT400088166*	*S. tuberosum*
StbZIP04	*PGSC0003DMT400007165*	*S. tuberosum*
StbZIP05	*PGSC0003DMT400085559*	*S. tuberosum*
StbZIP08	*PGSC0003DMT400079474*	*S. tuberosum*
StbZIP12	*PGSC0003DMT400040054*	*S. tuberosum*
StbZIP13	*PGSC0003DMT400009068*	*S. tuberosum*

Table S2. continued

Gene Name	Locus names or Accession nos. or AGI codes	Species
StbZIP17	*PGSC0003DMT400058187*	*S. tuberosum*
StbZIP26	*PGSC0003DMT400009493*	*S. tuberosum*
StbZIP31	*PGSC0003DMT400018832*	*S. tuberosum*
StbZIP43	*PGSC0003DMT400043542*	*S. tuberosum*
ZmbZIP9	*GRMZM2G073427*	*Zea mays*
ZmbZIP16	*GRMZM2G019446*	*Z. mays*
ZmbZIP17	*GRMZM2G016150*	*Z. mays*
ZmbZIP60	*GRMZM2G007063*	*Z. mays*
ZmbZIP65	*GRMZM2G098904*	*Z. mays*
ZmbZIP85	*GRMZM2G015534*	*Z. mays*
ZmbZIP112	*GRMZM2G103647*	*Z. mays*
ZmbZIP7	*GRMZM2G093020*	*Z. mays*
ZmbZIP22	*AC203957.3_FG004*	*Z. mays*
ZmbZIP59	*GRMZM2G361611*	*Z. mays*
ZmbZIP64	*GRMZM2G444748*	*Z. mays*
ZmbZIP87	*GRMZM2G092137*	*Z. mays*
PtobZIP42	*POTOM_026607*	*Populus tomentosa*
PtobZIP25	*POTOM_020647*	*P. tomentosa*
PtobZIP32	*POTOM_021892*	*P. tomentosa*
PtobZIP7	*POTOM_009536*	*P. tomentosa*
PtobZIP49	*POTOM_031535*	*P. tomentosa*
PtobZIP63	*POTOM_038208*	*P. tomentosa*
PtobZIP75	*POTOM_048317*	*P. tomentosa*
PtobZIP15	*POTOM_010759*	*P. tomentosa*
PtobZIP44	*POTOM_027938*	*P. tomentosa*
PtobZIP27	*POTOM_021242*	*P. tomentosa*
PtobZIP21	*POTOM_017716*	*P. tomentosa*
PtobZIP55	*POTOM_034782*	*P. tomentosa*
PtobZIP33	*POTOM_022015*	*P. tomentosa*
PtobZIP6	*POTOM_009428*	*P. tomentosa*

Table S3. The conserved motifs of the C/S1-bZIP proteins in Fig. 3.

Motif	Motif Consensus	Width
**Motif 1**	**VDERKRKRMJSNRESARRSRMRKQKHLDDLVTQVSQLRKENNQJLTRINV**	**50**
**Motif 2**	**TTQRYLNVEAENSILRAQIEELSHRLKSLNEILKYVNGSNG**	**41**
**Motif 3**	**ADDSFLNPLNLAYLNQPIMASADLFQY**	**27**
**Motif 4**	**MASSSGASSGSTKMQRSGSEE**	**21**
**Motif 5**	**CPNPHPQPLGRNLTAPIDSEEYRAFLKSKLDLACAAVAMSR**	**41**
**Motif 6**	**DGGSLGIPALPTAQKKQEVQARQTTSGSSREDSDDDDLEGDTGTNENMDP**	**50**
**Motif 7**	**ADDSFLNPLNLAYLNQPIMASADLFQY**	**26**
**Motif 8**	**FQNPWQVGAPVGPAGASPMFD**	**21**
**Motif 9**	**MNSVFSVDDFSGPFW**	**15**
**Motif 10**	**PSPGMPFVGGQVDASTDVAVP**	**21**

Table S4. Identification of uORFs in S1-bZIPs from different species

Protein Name	uORF sequence	length (aa)	Species
**AtbZIP1**	**MINLNQFLVYHSISVVILHWFYVIS**	**25**	***Arabidopsis thaliana***
**AtbZIP2**	**MTPVLCEILLSGLTVKSALCRRTHLVQSFSVVFLYWFYNVS**	**41**	***A. thaliana***
**AtbZIP11**	**MSPIILSEIFLSGFMLNSTIRRRTHLVQSFSVVFLYWLYYVS**	**42**	***A. thaliana***
**AtbZIP44**	**MSPVISEILRSGLTIDSSLRRRTHLVQSFSVVFLYWFYVFS**	**41**	***A. thaliana***
**AtbZIP53**	**MSYSILFRRIRILHSFSVVYLYYTYVFS**	**28**	***A. thaliana***
**MdbZIP2**	**–**	**–**	***Malus domestica***
**MdbZIP3**	**–**	**–**	***M. domestica***
**MdbZIP41**	**–**	**–**	***M. domestica***
**MdbZIP61**	**–**	**–**	***M. domestica***
**MdbZIP40**	**–**	**–**	***M. domestica***
**MdbZIP73**	**–**	**–**	***M. domestica***
**MdbZIP20**	**–**	**–**	***M. domestica***
**MdbZIP63**	**–**	**–**	***M. domestica***
**MdbZIP64**	**–**	**–**	***M. domestica***
**SlbZIP04**	**MIHMRRVRIMHSFSVVFLYWFYVFS**	**25**	***Solanum lycopersicum***
**SlbZIP07**	**MISAISITFLGDCFRQSFSVLFLYHFYDFS**	**30**	***S. lycopersicum***
**SlbZIP10**	**MINSTYRRRTHLVQSFSVVFLYWFYFIS**	**28**	***S. lycopersicum***
**SlbZIP15**	**–**	**–**	***S. lycopersicum***
**SlbZIP19**	**–**	**–**	***S. lycopersicum***
**SlbZIP34**	**–**	**–**	***S. lycopersicum***
**SlbZIP39**	**–**	**–**	***S. lycopersicum***
**StbZIP02**	**–**	**–**	***Solanum tuberosum***
**StbZIP04**	**–**	**–**	***S. tuberosum***
**StbZIP05**	**–**	**–**	***S. tuberosum***
**StbZIP08**	**–**	**–**	***S. tuberosum***
**StbZIP12**	**–**	**–**	***S. tuberosum***
**StbZIP13**	**–**	**–**	***S. tuberosum***
**StbZIP17**	**–**	**–**	***S. tuberosum***
**StbZIP26**	**–**	**–**	***S. tuberosum***
**StbZIP31**	**–**	**–**	***S. tuberosum***
**StbZIP43**	**–**	**–**	***S. tuberosum***
**ZmbZIP7**	**MSQILSEVSHRCRFMINSTLRRGTHLVLSFSVVFLYWFYVFSVRS**	**45**	***Zea mays***
**ZmbZIP22**	**MLLLLVLSEHLLLSGFMIGSAITRLVQSFSVVFLYWFYVFSVSCHCQ**	**46**	***Z. mays***
**ZmbZIP59**	**–**	**–**	***Z. mays***
**ZmbZIP64**	**–**	**–**	***Z. mays***
**ZmbZIP87**	**–**	**–**	***Z. mays***
**PtobZIP49**	**MKYFRLFCQIKISHSVSVVLLYWFYVFS**	**28**	***Populus tomentosa***
**PtobZIP63**	**MNHLRLFCRNKISHSVSVVLLYWFYVFS**	**28**	***P. tomentosa***
**PtobZIP75**	**MTYSILMRRIRILHSFSVVFLYWFYVFS**	**41**	***P. tomentosa***
**PtobZIP15**	**–**	**–**	***P. tomentosa***
**PtobZIP44**	**MTPVLSEILLSGFTINSTLRRGTHLVQSFSVVFLYWFYVFS**	**41**	***P. tomentosa***

Table S4. continued

Protein Name	uORF sequence	length (aa)	Species
**PtobZIP27**	**–**	**–**	***P. tomentosa***
**PtobZIP21**	**–**	**–**	***P. tomentosa***
**PtobZIP55**	**MSPILSEIFISGCMINSTFRRRTHLVQSFSVVFLYWLYYVS**	**41**	***P. tomentosa***
**PtobZIP33**	**–**	**–**	***P. tomentosa***
**PtobZIP6**	**–**	**–**	***P. tomentosa***

Table S5. Primer sequences used in this study.

Name	Forward (5'- 3')	Reverse (5'- 3')	Applications
PtobZIP42	GGAGCAGAAAGCAGGAGGAA	CCCGAGTTTTGTTGACAGGG	
PtobZIP25	GACCAATCCTGGTGTCTAG	GCATGGCATTATCTTTATAATT	
PtobZIP32	TCCAAATCCTCATCCGCAGC	CCATGACCAGTGCGAAAAGC	
PtobZIP7	ACCAATGCTGTAAAGCCGGA	CCCGCTTTGTGCCATTGAAA	
PtobZIP49	ATCAGGTTCTGACGTTGATGCG	TGTTCTGTGATTCGAGAGC	
PtobZIP63	TCAGGCTCTGAAATTGACCCA	AACTTTGTTGTCTGATTCGAGAG	
PtobZIP75	TGTGGAATTGACTGAGAGGC	AATAGGCTGTACTGAACACG	
PtobZIP15	GGTCGTTGAACTCTGTGTTGC	TTGAAACATATCAGCAGATGCC	RT-qPCR
PtobZIP44	CGAAGCTGAGAACTCTATCC	CAAAAGCACCAGTAGTGGG	
PtobZIP27	GAGATCCTGACAAGGATGA	TAGTCGCAGAAACAGAGGC	
PtobZIP21	GCGTTACTTGAGTGTTGAGG	ATTGAAGGTGATCGAGTCTC	
PtobZIP55	AGGCCGATAACTCAATCTTG	CATTAAAGATGCTTGAATCTCC	
PtobZIP33	CCATCAATGTCACAACACAGC	ATCAGGGAGATCCTCGTGATG	
PtobZIP6	GTCACAACACAGCACTTCTT	GCACTAGTTTGGAGATCCTC	
PtoUBQ10	GTTGATTTTTGCTGGGAAGC	GATCTTGGCCTTCACGTTGT	
pGADT7-*PtobZIP42*	GGAGGCCAGTGAATTCATGGAGGAGCAGAAAGCAGG	TCTGCAGCTCGAGCTCTTAAGACCAAATGTTTGTTATAC AGC	
pGADT7-*PtobZIP25*	GGAGGCCAGTGAATTCATGTCACACAGACCAATCCT	TCTGCAGCTCGAGCTCTTACGACTGCTCCCCATTGG	
pGADT7-*PtobZIP32*	GGAGGCCAGTGAATTCATGAACAGTGTGTTCTCAGT	TCTGCAGCTCGAGCTCTCATTTCTTTTTGCCATCC	Yeast two-hyrid assay
pGADT7-*PtobZIP7*	GGAGGCCAGTGAATTCATGAATAGTGTCTTCTCAGTGG	TCTGCAGCTCGAGCTCTCATTTCTTTTTGGCATCCCTCG	
pGBKT7-*PtobZIP49*	ATGGAGGCCGAATTCATGTCATCATCCCTGGCAAAG	CCGCTGCAGGTCGACTCTAGAATTTGAACATCCCAGAA GC	

Table S5. continued

Name	Forward (5'- 3')	Reverse (5'- 3')	Applications
pGBKT7-*PtobZIP63*	ATGGAGGCCGAATTCATGCCACCATCCTTTGCAA	CCGCTGCAGGTCGACTCTAAACTTTGAACATCCCAG	Yeast two-hyrid assay
pGBKT7-*PtobZIP75*	ATGGAGGCCGAATTCATGTCTGCAAGGCAAGCAGCGA	CCGCTGCAGGTCGACTTCAGCAGATGTCATAATAGGCTGT	
pGBKT7-*PtobZIP15*	ATGGAGGCCGAATTCATGTCTGCAAGGCAAGCGG	CCGCTGCAGGTCGACTCTAATATTGAAACATATCAGCAGAT	
pGBKT7-*PtobZIP44*	ATGGAGGCCGAATTCATGGCCTCTTCTAGTGGGGCATC	CCGCTGCAGGTCGACTTAGTACATGATCATGTCCATG	
pGBKT7-*PtobZIP27*	ATGGAGGCCGAATTCATGGCCTCTTCTAGTGGGGCATC	CCGCTGCAGGTCGACTCAGTACATAACCATGTCC	
pGBKT7-*PtobZIP21*	ATGGAGGCCGAATTCATGGCTTCCTCTAGTGGAAC	CCGCTGCAGGTCGACTCAGTAATGAAACATCTCTGC	
pGBKT7-*PtobZIP55*	ATGGAGGCCGAATTCATGGCTTCGTCCAGTGGAAC	CCGCTGCAGGTCGACTCAGTATTGAAACATATCT	
pGBKT7-*PtobZIP33*	ATGGAGGCCGAATTCATGGCATCCTCCAGTGGGGATTCC	CCGCTGCAGGTCGACTCAATACTGAAACAAATCTGGG	
pGBKT7-*PtobZIP6*	ATGGAGGCCGAATTCATGGCATCCTCTAGTGGGAATT	CCGCTGCAGGTCGACTTCAATACTGAAACAAATCTGGAGATG	
amiRNA-1	CTGCAAGGCGATTAAGTTGGGTAAC	GAATTCTCAATCTAGACTTGTTCTCTACATATATATTCCT	Construction of transgenic vectors
amiRNA-2	GAGAACAAGTCTAGATTGAGAATTCACAGGTCGTGATATG	GAGAGCAAGTCTAGAATGAGAAATCAAAGAGAATCAATGA	
amiRNA-3	GATTTCTCATTCTAGACTTGCTCTCTCTCTTTTGTATTCC	GCGGATAACAATTTCACACAGGAA ACAG	
amiRNA-4	CTGCAAGGCGATTAAGTTGGGTAAC	GCGGATAACAATTTCACACAGGAA ACAG	
bZIP55/21-amiRNA-G	CCCAAAGAGCTCTTCTTCTTC	GAGTGGTGATATTGATGCTGG	The selection of transgenic lines
