# Supplementary figures and images for: Genome-Wide Analysis of C/S1-bZIP Subfamilies in Populus tomentosa and Unraveling the Role of PtobZIP55/21 in Response to Low Energy
Source: Int J Mol Sci. 2024 May 9;25(10):5163. doi: 10.3390/ijms25105163 (PMC11120861; doi:10.3390/ijms25105163)

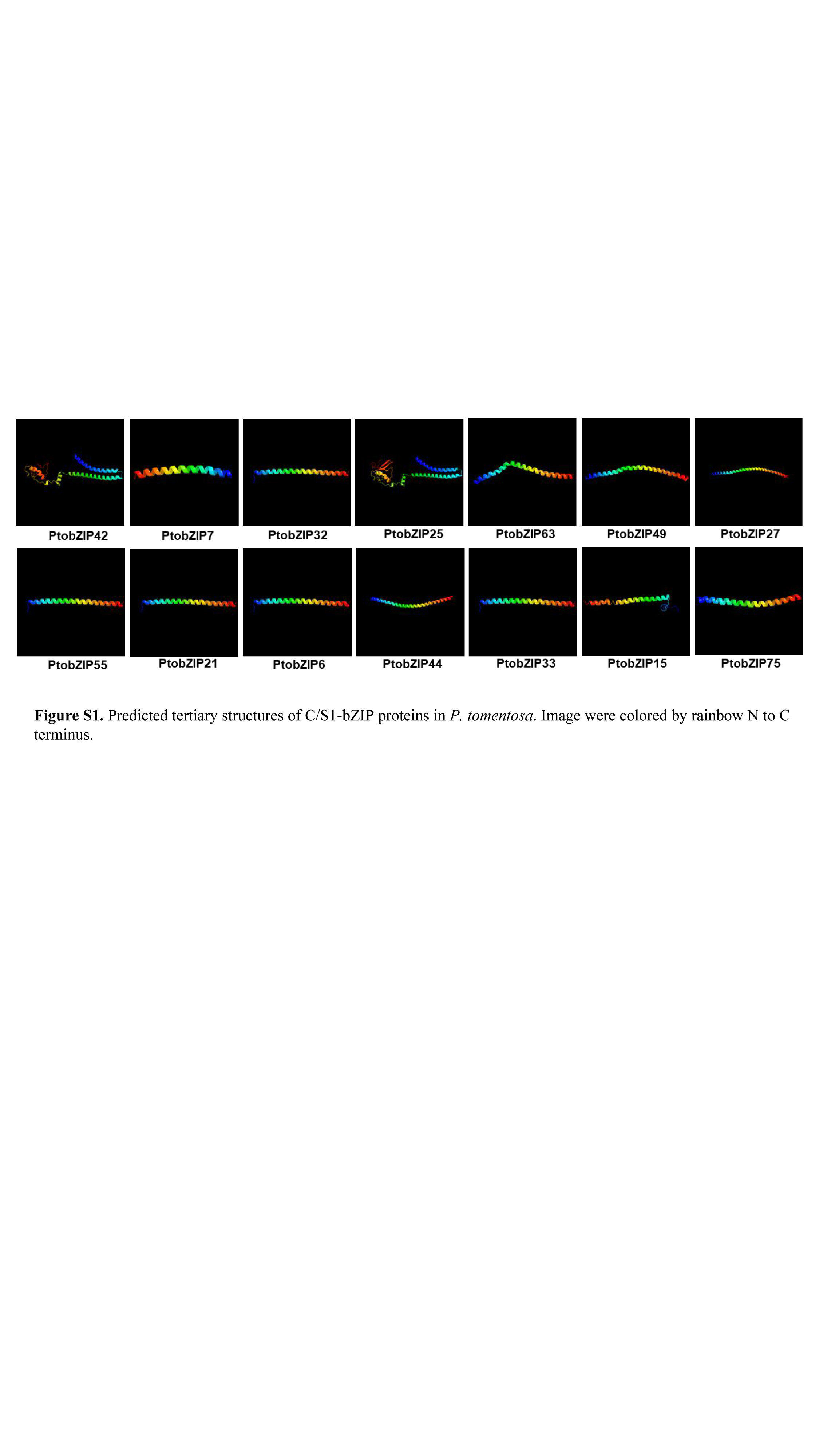

Supplement: Supplementary file 1 [file ijms-25-05163-s001.zip › ijms-2971733-supplementary/Figure S1.tif]

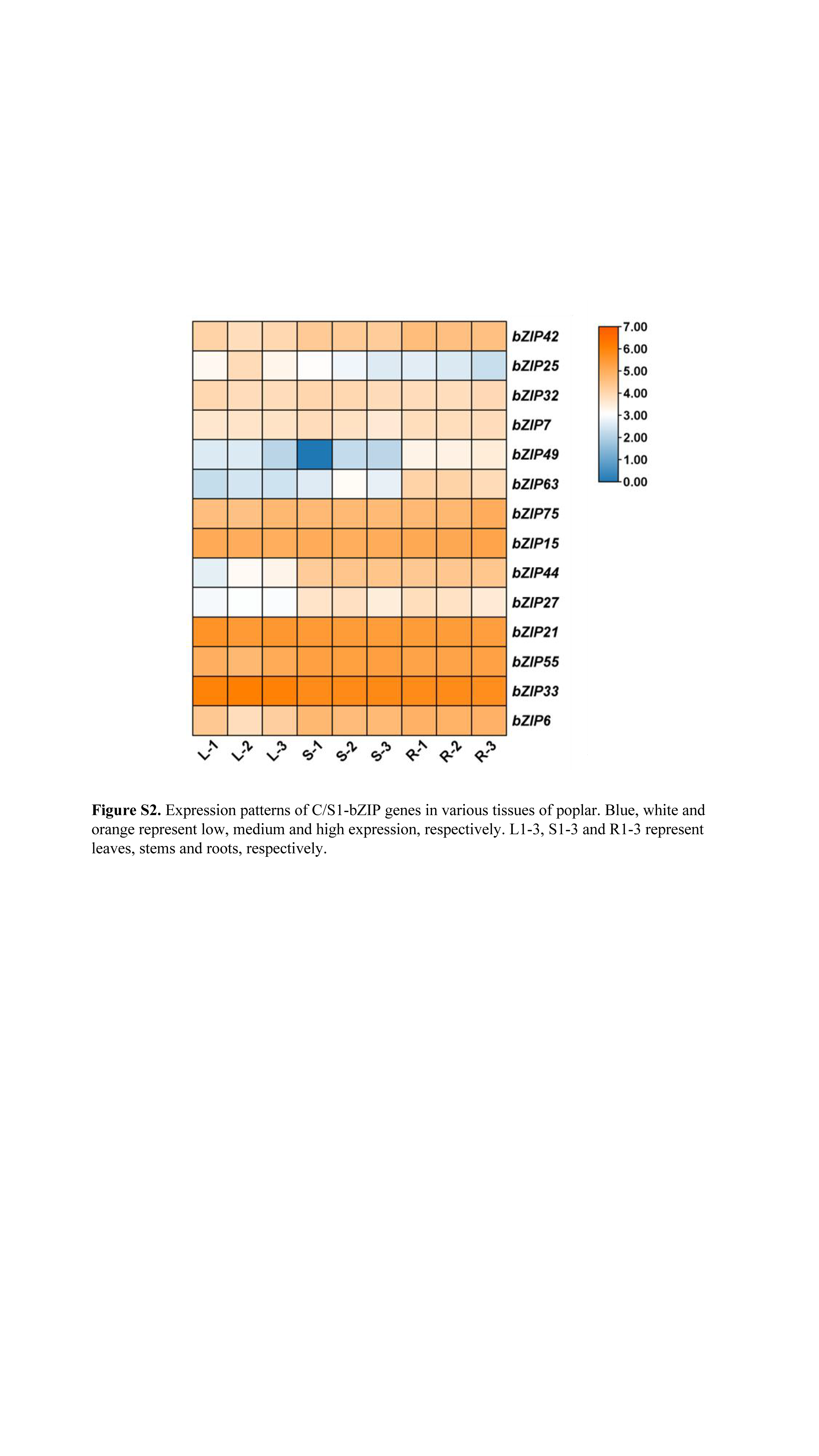

Supplement: Supplementary file 1 [file ijms-25-05163-s001.zip › ijms-2971733-supplementary/Figure S2.tif]

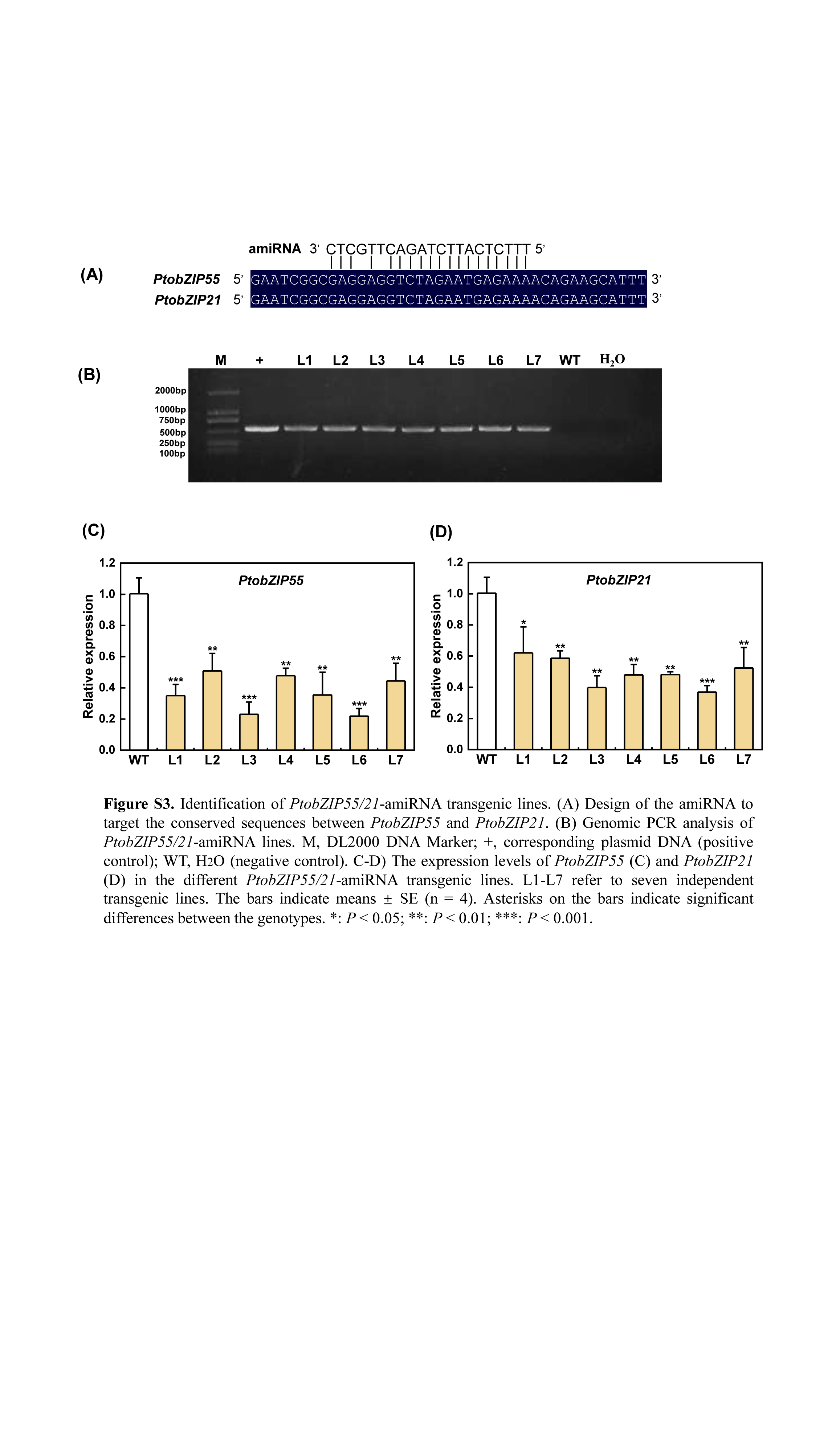

Supplement: Supplementary file 1 [file ijms-25-05163-s001.zip › ijms-2971733-supplementary/Figure S3.tif]
